# ImpRess: an Implementation Readiness checklist developed using a systematic review of randomised controlled trials assessing cognitive stimulation for dementia

**DOI:** 10.1186/s12874-016-0268-2

**Published:** 2016-12-01

**Authors:** Amy Streater, Aimee Spector, Elisa Aguirre, Jacki Stansfeld, Martin Orrell

**Affiliations:** 1 North East London Foundation Trust, London, UK; 2 University College London, London, UK; 3 University of Nottingham, London, UK

**Keywords:** Cognitive stimulation, Cognitive Stimulation Therapy (CST), Dementia, Implementation, Implementation Readiness (ImpRess), Reporting

## Abstract

**Background:**

Research reporting results of clinical trials, psychosocial or technological interventions frequently omit critical details needed to inform implementation in practice. The aim of this article is to develop an Implementation Readiness (ImpRess) checklist, that includes criteria deemed useful in measuring readiness for implementation and apply it to trials of cognitive stimulation in dementia, providing a systematic review of their readiness for widespread implementation.

**Methods:**

Five electronic databases were searched. After initial screening of papers, two reviewers assessed quality and scored the included studies based on the ImpRess checklist specifically developed for this review.

**Results:**

Twenty studies met the inclusion criteria. As determined by the ImpRess checklist, scores ranged from 11 to 29 out of 52. According to the checklist the most comprehensive and ready to implement version of cognitive stimulation was Cognitive Stimulation Therapy.

**Conclusions:**

Reports of interventions rarely include consideration of implementation in practice. Contrary to the growing number of reporting guidelines, crucial items within the ImpRess checklist have been frequently overlooked. This study was able to show that the ImpRess checklist was feasible in practice and reliable. The checklist may be useful in evaluating readiness for implementation for other manualised interventions.

## Background

There has been a rapid growth in the number of evidence based psychosocial interventions for dementia. There are however, concerns that very few of these get in to practice, even when there is good evidence in benefits for the service user. This may be because studies do not consider all the necessary factors to promote implementation in practice. Cognitive stimulation is defined as ‘engagement in a range of activities and discussions (usually in a group) aimed at general enhancement of cognitive and social functioning’ [1]. Cognitive stimulation is considered to have the ‘strongest evidence by far’ [2] for cognitive benefit, with the National Institute for Health and Care Excellence (NICE) guidelines (2006) recommending its use for all people with mild to moderate dementia [3]. However, evidence in relation to its implementation readiness and dissemination of this is currently lacking.

The Medical Research Council (MRC) framework for complex interventions (2008) places emphasise on the implementation phase of interventions to demonstrate the applicability of an intervention in real life settings [4]. Adherence to the framework would assist in identifying the usefulness of the intervention, how it works, and should provide enough detail so that the implementation phase of the framework can be explored fully. This would further assist the use of psychosocial therapies in practice in a timely manner. Process evaluation incorporates context, implementation, and mechanisms of impact as key components in the design and testing of complex interventions [5]. This broader approach is especially relevant due to the well-documented gap between research findings and their application in practice [6].

A systematic review was carried out to identify studies on cognitive stimulation. We then developed an easy to use, comprehensive checklist to assess the level of reporting in randomised controlled trials (RCTs) reporting on cognitive stimulation studies to determine the RCTs level of readiness for implementation in practice.

## Methods

### Criteria for selecting studies

A systematic search was carried out and included studies were RCTs that assessed the effectiveness of cognitive stimulation for people with dementia in a variety of care settings. RCTs were chosen as the only acceptable design for included studies because it is considered the gold standard when assessing the effectiveness of an intervention. All studies; (i) met the definition of cognitive stimulation [1], (ii) were published in a peer reviewed journals and were written in English, (iii) had a researcher, staff member or family caregiver deliver the intervention to people with dementia, (iv) demonstrated significant beneficial effects on cognition and/or behaviour for the person with dementia, (v) included participants with a dementia diagnosis, (vi) received the intervention for a minimum of four weeks with no restrictions placed regarding its duration, and (vii) had outcomes looking at performance on at least one psychometrically sound test of cognitive functioning. The inclusion criteria were lifted from a previously conducted Cochrane review [7].

### Development of checklist

The checklist was derived from a set of criteria developed to appraise the quality of reporting of implementation of workplace interventions [8]. The original checklist had one question per each of the ten themes but this was considered insufficient to provide a comprehensive guide to potential barriers to implementation. So, the newly devised ImpRess checklist was developed, guided by the MRC framework (2008) [4] to incorporate questions relevant to implementation. Considerations raised in this framework include; neglecting the development stages, over emphasis on the evaluation stage, and a lack of practical consideration in relation to implementation. Consequently, we worked collaboratively to consider each of the ten themes and what was deemed important when considering the implementation readiness of cognitive stimulation. We have extensive experience in the development, set up, delivery and evaluation of Cognitive Stimulation Therapy (CST) and considered that their practical knowledge in the delivery of the programme was useful in developing the ImpRess checklist. The intention was to pragmatically consider what would be expected and useful in the reporting of published papers.

As a result, the 26-question checklist was developed covering 10 themes including; motivation, theory of change, implementation context, experience, planning consultations, delivery collaborations, manager support, employee support, resources, and population characteristics and each theme included sub-questions (Table 1).

**Table 1:** Implementation Readiness (ImpRess) checklist

Number	Theme	Original checklist question	Number	ImpRess question
1	Motivation (max score 10)	Does the study describe why management decided to subject the employee population to the organisational change?	1	Does the existing evidence suggest the intervention is likely to be cost effective?
			2	Does the existing evidence suggest the intervention is likely to be effective for the primary outcome?
			3	Does the existing evidence suggest the intervention is likely to be effective for other key outcomes?
			4	Are there other benefits for the patient (qualitative)?
			5	Are there benefits for the organisation?
2	Theory of change (max score 8)	Was the intervention design influenced by a theory of change describing the proposed pathway from implementation to health outcome?	6	Are the outcomes clearly defined?
			7	Is how the intervention works clearly defined?
			8	Is the design suitable for the kind of intervention (RCT)?
			9	Is there a coherent theoretical base?
3	Implementation context (max score 4)	Does the study provide any useful contextual information relevant to the implementation of the intervention?	10	Is the intervention standardised?
			11	Can it be widely implemented in to practice (following on from a research setting)?
4	Experience (max score 4)	Does the study establish whether those implementing the intervention had appropriate experience?	12	Is the skills and experience of the person delivering the intervention clearly described?
			13	Is there monitoring of the delivery (attendance/adherence) of the intervention?
5	Planning consultations (max score 4)	Is there a report of consultation/collaboration processes between managers, employees and any other relevant parties during the planning stage?	14	Is the amount of time necessary to set up the intervention specified?
			15	Is the planning and setting up of the sessions clearly defined?
6	Delivery collaborations (max score 4)	Is there a report of consultation/collaboration processes between managers, employees and any other relevant parties during the delivery stage?	16	Does it specify the amount of time required for each session and for the duration of the programme?
			17	Are the potential and facilitator barriers to the delivery of the intervention described?
7	Manager support (max score 2)	Were on-site managers/supervisors supportive of the intervention?	18	Is the level of managerial support described during the intervention/evaluation?
8	Employee support (max score 2)	Were employees supportive of the intervention?	19	Is the level of support required by staff members to deliver the intervention described?
9	Resources (max score 10)	Does the study give information about the resources required in implementing the intervention?	20	Are the resources required to deliver the intervention specified?
			21	Is the training costs specified?
			22	Are the training materials specified?
			23	Are there manuals for the intervention?
			24	Are the materials easy to source?
10	Population characteristics (max score 4)	Does the study provide information on the characteristics of the people for whom the intervention was beneficial, and the characteristics of those for whom it was harmful or ineffective?	25	Are the population characteristics specified?
			26	Does it specify who benefits most from the intervention?

Each theme appraised the level of information reported in the published paper to assess ‘readiness for implementation’, which encompasses factors related to implementation of cognitive stimulation in practice. Each question required a yes or no response and scored accordingly, with total score ranging from zero to 52.

### ImpRess checklist outcome measures

The ImpRess checklist comprises of 26 questions across 10 themes with zero given when no information was provided, one point if partially answered and two points if the question is fully answered. There was no weighting given to a particular theme and the overall score was used to determine implementation readiness.

#### Motivation

Motivation to use the intervention attempts to identify cost effectiveness (Q1) and includes the expected beneficial effects in outcomes for the person with dementia and organisation (Q2 & 3) to demonstrate a motivational aspect to the delivery of the intervention (e.g. a significant effect for the person with dementia). ‘Motivation’ also includes the presentation of qualitative information in focus group or individual interviews (Q4) gathered on the intervention to ensure the reporting of benefits for the person with dementia or staff member, in addition to organisational benefits (Q5) as identified by recommendations and guidelines included in the paper.

#### Theory of change

Includes the description of any outcomes used (Q6), model of how the intervention works (Q7), study design (Q8), and theoretical base (Q9). The description of chosen outcomes is used to identify what is being measured, how it is being measured, and difference between time points. ‘Theory of change’ includes a definition of how the intervention works (regarding mechanisms of change) and determining the suitability of the study design with a focus on how theory has been applied to the research and to justify the use of the intervention.

#### Implementation context

Is the standardised nature of the intervention and its reproducibility in a real life setting (Q10 & 11). To determine standardisation of the intervention, the study requires details relating to the type and delivery of the intervention, resources, and type of person required to deliver the intervention. This theme relates to the consideration of the large-scale dissemination of the intervention.

#### Experience

Identifies the level of experience required by the person to deliver the intervention (Q12) and includes the recording of attendance and adherence to the intervention (Q13).

#### Planning consultations

Identifies the level of information detailed within the study in the setting up and planning of the intervention and sessions (Q14 & 15). This theme is scored on whether the time taken to set up the programme is mentioned and planning issues are mentioned.

#### Delivery collaborations

This includes the time requirements to deliver the intervention and identify any potential barriers (Q16 & 17). This includes the amount of time necessary to run the session, overall programme plan, potential or lack of barriers related to the intervention, and difficulties a person might experience when delivering the therapy.

#### Manager support

Enables the identification of the level of support required within the workplace in order to implement the therapy and considers the logistics and ease of implementation of an intervention (Q18).

#### Employee support

Level of ‘Employee support’ is useful when considering implementation into practice (Q19) as it may affect the intervention. For instance, the level of training or number of staff required to deliver the intervention may impact on the ease in which the cognitive stimulation programme can be implemented in the care setting.

#### Resources

Training in psychosocial interventions can be hard to access, with many interventions having no training manual or being so poorly specified that the intervention could not be reliably replicated in practice [9]. ‘Resources’ as a theme identifies any reference to resources required to deliver the intervention, the cost of training, training materials (e.g. DVD), manuals, and the sourcing of materials (Q20, 21, 22, 23 & 24).

#### Population characteristics

Provides insight into whom the intervention is for (Q25) and who benefits most (Q26). For a full score to be awarded for the description of the population characteristics, the following details were required: (1) type of dementia, (2) exclusion criteria, (3) level of impairment, (4) intervention setting, (5) age range, (6) gender, (7) ethnicity, and (8) country of residence. These details were considered necessary, in addition to who benefited most, to give a comprehensive account of the intended target population.

### Data sources

PsycINFO, Medline, EMBASE, CINAHL and SCOPUS were searched between January 2011 and April 2013. Search terms were matched to a Cochrane review on cognitive stimulation [7] and prior to these search dates, the included papers in the Cochrane review were used for this review. The search terms used for cognitive stimulation were: ‘cognitive stimulation’, ‘reality orientation’, ‘memory therapy’, ‘memory groups’, ‘memory support’, ‘memory stimulation’, ‘global stimulation’, ‘cognitive psychostimulation’.

### Study selection

A researcher independently screened by title and abstract of retrieved studies, and the full text of potentially eligible studies. At the screening stage any discrepancies were resolved via discussion between two researchers. One researcher carried out data extraction using the ImpRess checklist. Another researcher independently scored the papers and results were compared, there was 99.4% inter-rater reliability.

## Results

### Overview of search hits and included studies

**Fig. 1:**
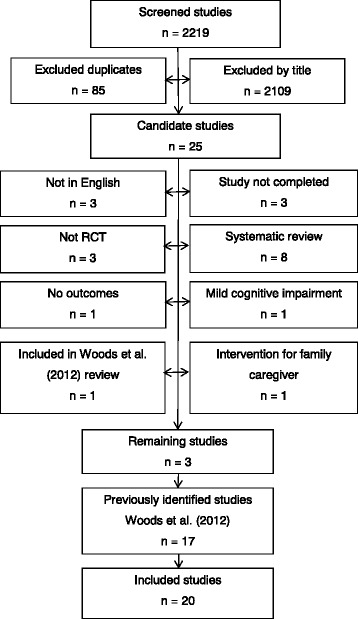
Consort diagram of included studies

The electronic databases were searched and screened by title and abstract. After initial screening studies were then further reviewed to ensure they met the inclusion criteria. A total of 20 papers were included in this review, of which 17 had been identified in the previous review [8] and three were new (Fig. 1). The ImpRess checklist was applied to the 20 included studies. Studies were scored two points if they fully answered the question, one point if partially answered, and a score of zero if the question was unanswered (Table 2).

**Table 2:** Implementation Readiness (ImpRess) checklist score for included studies

Studies	Theme										
	Motivation	Theory of change	Implementation context	Experience	Planning consultations	Delivery collaborations	Manager support	Employee support	Resources	Population characteristics	Total
Akanuma et al., 2011 [21]	4	7	4	2	0	2	0	2	0	2	23
Baines et al., 1987 [22]	3	7	4	4	0	3	2	2	5	2	32
Baldelli et al., 1993 [23]	2	5	1	1	0	2	0	0	0	2	14
Baldelli et al., 2002 [10]	2	4	1	0	0	2	0	0	0	2	11
Bottino et al., 2005 [24]	2	7	3	0	0	2	0	0	0	2	16
Breuil et al., 1994 [25]	0	6	3	2	0	3	0	0	1	3	18
Buschert et al., 2011 [26]	6	7	4	2	1	3	0	0	2	2	27
Chapman et al., 2004 [11]	4	7	4	3	0	3	0	1	5	2	29
Coen et al., 2011 [27]	7	4	3	3	0	3	0	2	3	2	27
Ferrario et al., 1991 [28]	1	5	1	1	0	3	0	0	0	1	12
Gerber et al., 1991 [29]	0	5	4	0	0	2	0	0	1	2	14
Graessel et al., 2011 [30]	2	8	4	1	1	2	0	1	2	2	23
Hanley et al., 1981 [31]	2	5	3	3	0	2	0	2	4	2	23
Maci et al., 2012[32]	4	5	4	2	0	2	0	2	1	2	22
Onder et al., 2005 [33]	4	5	4	2	0	3	0	2	4	2	26
Requena et al., 2006 [34]	1	7	3	0	0	2	0	0	1	2	16
Spector et al., 2001 [35]	2	7	4	2	2	4	0	2	4	1	28
Spector et al., 2003 [12]	2	7	4	2	0	4	0	2	4	4	29
Wallis et al., 1983 [36]	2	5	3	3	0	2	0	2	1	2	20
Woods et al., 1979 [37]	2	6	3	3	0	3	0	2	5	2	26

### Motivation

There was a lack of information across the included papers detailing the different aspects of ‘Motivation’. Only one paper included information relating to the cost effectiveness of the intervention in a formal capacity by referencing an additional paper on economic analysis. All papers, excluding three, provided sufficient detail evidencing the choice of primary outcomes and in relation to evidence suggesting effectiveness for other key outcomes seven studies provided supporting evidence. Only three studies provided evidence of qualitative benefit and two studies highlighted organisational benefit.

### Theory of change

All studies reported on the outcome measures used to assess the participants, but the description varied in length and level of detail. Only two studies scored a maximum score, 16 studies were assigned one point for providing partial details on the outcome measures being used, and one study was assigned a zero for not clearly defining the outcome measures. The majority of studies clearly defined how the intervention worked except for five studies that omitted this information. Being RCTs, all included studies had a suitable design.

### Implementation context

All studies provided a clear explanation of a standardised intervention. Seven studies went further by providing sufficient detail for the intervention to be implemented to some degree into practice, and 10 studies provided enough detail for the intervention to be fully put into practice.

### Experience

Ten studies sufficiently detailed the level of experience required to deliver the intervention. Seven studies provided no further information about the person so a partial score was given. Only two studies provided full details of attendance and adherence and a partial score was assigned to seven studies for providing details relating to drop outs inferring that records of attendance were kept. In particular, one study detailed weekly meetings to ensure the intervention was delivered as designed.

### Planning consultations

Details on ‘Planning consultations’ were lacking across studies. One study made reference to the small amount of time necessary to set up the intervention, whilst two studies provided details regarding the planning of the session structure. Aside from these aforementioned studies, no details were provided concerning the consultation and planning necessary to deliver the intervention.

### Delivery collaborations

All studies provided full details on the delivery frequency of the intervention. Just over half of the studies highlighted potential barriers to the facilitation of the programme, such as feasibility of a long-term intervention, time commitment, difficulty in scheduling sessions, intervention not meeting staff expectations, and demands put on staff.

### Manager support

Only one study made reference to ‘Manager support’ by providing positive feedback following the intervention in regards to rescheduling meetings and the intervention continuing after the research involvement. No other study made reference to manager involvement.

### Employee support

Ten studies provided information on the required level of ‘Employee support’. However, a partial score was assigned to two studies for mentioning staff or aide, and family carer without providing further detail.

### Resources

Very few studies provided a comprehensive report of the resources required to deliver the intervention. Six studies detailed the materials required (e.g. blackboard, clock), and numerous studies detailed some form of training received by the person delivering the intervention. Despite this, there was no mention of training costs associated with this. The training materials varied across the studies from demonstrations, discussion, handouts, videos and role-play and six of the 20 included studies made reference to a manual. Three studies received a full score and three a partial score, as sourcing of the manual was not provided. Only six studies provided information relating to the sourcing of materials to run the programme.

### Population characteristics

All the included studies provided information regarding the population characteristics that the intervention was targeting. However, two studies were assigned one point due to missing some of the criteria. Only two studies made reference to who benefited most from the intervention.

### ImpRess scoring

A total score of 52 could be attained for each included study. After applying the ImpRess checklist, the scores ranged from 11 [10] to 29 [11, 12]. Aside from reporting alone, a pragmatic decision was made to identify the same intervention being reported to evaluate the most recent paper for readiness for implementation. This was considered a logical step as it was expected that references would be made to the original research in the most up to date published version of the intervention. The version of cognitive stimulation that scored most highly had three papers that were included in the systematic literature search. This demonstrated that the more evidenced based version of cognitive stimulation was CST [12] as this particular programme scored higher on implementation readiness.

## Discussion

This is the first systematic review to apply the newly devised ImpRess checklist to cognitive stimulation in dementia. This review demonstrated an average 43% reporting rate when applied across the 20 included studies. Falling below 50% in the reporting of readiness for implementation indicated the lack of reporting, including information related to getting the intervention in to practice. There was a higher reporting rate for ‘Implementation context’, ‘Theory of change’, and ‘Delivery collaborations’. Arguably, this is to be expected due to the standard format in the reporting of studies as items such as outcome measures and standardisation of the intervention should be adequately reported.

Specifically, it is clear that the pragmatic considerations are missing and highlighted in the lack of reporting when considering implementation in practice, in particular to ‘Planning consultations’, ‘Manager support’, and ‘Resources’. Nonetheless, the included studies were carried out over a 34-year period and yet, there was no increase in the reported scores over this timeframe. This was an unexpected finding as it was assumed that more recent studies would score higher due to the recent emphasis on implementation science, the number of reporting frameworks, and the advances in psychosocial interventions, specifically in dementia.

Given that the reported interventions might not be at the implementation stage and undergone widespread dissemination the calculated scores are not wholly unexpected. Some reporting, such as qualitative benefits, might be expected at a later stage of dissemination. However, an improvement in the reported delivery of studies is a necessary step to enable implementation by providing healthcare professionals with an overview of the justification for, set up, delivery and evaluation of the intervention in one paper.

Overall, the ImpRess checklist provided a useful insight into the themes that tend to be overlooked in study reporting. The newly devised checklist may illustrate the broader context surrounding the programme and focus on specific factors needed for successful implementation. As most studies omitted information in relation to ‘Planning consultations’, this limits its application due to potential cost and practical implications in the delivery of the intervention. In addition, the reporting of ‘Manager support’ is advantageous to the successful implementation in the workplace. The reporting of ‘Resources’ across studies was minimal. No studies reported on training costs to deliver the cognitive stimulation programme. If training was required prior to the implementation of the intervention this could act as a restriction due to the associated costs and time constraints. In addition, a lack of information regarding training materials limits the ease to which the reported intervention can be implemented into practice.

A number of frameworks have shifted the focus to implementation and as a result there are several number of frameworks available but each have their own limitations. The Promoting Action on Research Implementation in Health Services (PARIHS) framework [13] identifies three key interacting elements that impact on successful implementation: Evidence, Context, and Facilitation. However, this framework tends to be utilised post hoc, instead of in the prospective stage of developing an intervention [14].

An alternative to the PARIHS framework is the Reach, Efficacy, Adoption, Implementation, and Maintenance (RE-AIM) framework that considers the individual and organisational factors that may impact on the delivery of an intervention [15]. A recent systematic review [16] applied the RE-AIM framework to implementation of psychosocial interventions in residential dementia care and identified ‘Adoption’ and ‘Maintenance’ as receiving less attention than the other items. The ‘implementation error’ [17] could explain the low treatment fidelity that may be improved upon if using and reporting the ‘Maintenance’ stage of the RE-AIM framework.

The Consolidated Framework for Implementation Research (CFIR) is another framework that includes five main domains; intervention characteristics, outer and inner settings, individual characteristics of those involved, and the implementation process [18]. The CFIR framework specifies a list of constructs that through a review of theories has generated general domains considered to influence implementation. Although comprehensive, this framework is an exhaustive list of potential areas of implementation that may hinder its use.

More recently the template for intervention description and replication (TIDieR) checklist and guide was published [19] and is similar in design to the ImpRess checklist but ignores the necessary outcome measures that might be deemed important when considering why a person or organisation might implement the intervention in the first place. Additionally, the TiDieR checklist is a descriptive checklist as opposed to the ImpRess checklist, which is designed to be a quick to complete indicator of an interventions readiness for implementation.

There was high inter-rater reliability (99.4%), however further research could involve a more detailed psychometric assessment of the ImpRess checklist and also using it to assess implementation readiness for other interventions. There are however, limitations to the ImpRess checklist. It cannot indicate the restrictions placed on the authors when submitting research for publication e.g. word count. In addition, if the research is looking at the effect of the intervention on participants, in this instance people with dementia, it may be deemed unnecessary to include additional information relating to staff. Information was difficult to extract from the text and left the answers for some of the questions in the ImpRess checklist open to interpretation. In regard to the cost effectiveness and recommendations of cognitive stimulation, these were only identified in 2006 [3, 20] so the reporting of this information within papers would not have been possible prior to this date. Including RCTs only is a limitation of this review and including cited or referenced papers to minimise the risk of missing this information would extend the scope of the ImpRess checklist.

## Conclusions

The ImpRess checklist could provide a useful tool for researchers and healthcare professionals to determine if an intervention, such as cognitive stimulation is suitable in their workplace based on information provided in a published paper.

This systematic review highlights the lack of information supplied in publications for further dissemination into practice. Consideration of the dissemination and long-term implementation of cognitive stimulation provides an opportunity to understand the barriers and pragmatic reasons for a lack of uptake of the therapy. If information is not included in the reported paper, it serves the purpose of highlighting where attention should be placed.

In order to effectively use the ImpRess checklist to determine how far an intervention is ready for implementation in practice, it is useful to bring together the specific group of papers covering the most comprehensive picture available. This may include papers on development work, economic analysis, and implementation studies. CST [12] has been rigorously evaluated and the focus is now on reporting the implementation of CST in practice [38, 39] to better understand the impact on the delivery of the programme and outcome measures for people with dementia.

There are currently no set guidelines on how to increase the uptake of psychosocial interventions in practice. However, the ImpRess checklist can go some way in promoting and aiding the reporting of evidence based practice, such as cognitive stimulation. Until a consensus is reached as to the level of information required when detailing an intervention, there is likely to be a gap between research and successful implementation in practice.

## Abbreviations

CFIR framework: Consolidated Framework for Implementation Research framework
CST: Cognitive Stimulation Therapy
ImpRess: Implementation Readiness
MRC: Medical Research Council
NICE: National Institute for Health and Care Excellence
PARIHS framework: Promoting Action on Research Implementation in Health Services framework
RCTs: Randomised Controlled Trials
RE-AIM framework: Reach, Efficacy, Adoption, Implementation, and Maintenance framework
TIDieR checklist: Template for intervention description and replication checklist
